# Levetiracetam attenuates diabetes-associated cognitive impairment and microglia polarization by suppressing neuroinflammation

**DOI:** 10.3389/fphar.2023.1145819

**Published:** 2023-05-04

**Authors:** Yun-yun Zhang, Lu Wang, Hua Guo, Ting-ting Han, Yan-hua Chang, Xiao-chuan Cui

**Affiliations:** ^1^ Department of General Practice, The Affiliated Wuxi People’s Hospital of Nanjing Medical University, Wuxi, Jiangsu, China; ^2^ Wuxi Medical Center, Nanjing Medical University, Wuxi, Jiangsu, China; ^3^ Department of Pathology, The Affiliated Wuxi People’s Hospital of Nanjing Medical University, Wuxi, Jiangsu, China

**Keywords:** diabetic, cognition, levetiracetam, microglia polarization, JNK/MAPK/ NF-κB

## Abstract

**Introduction:** Cognitive impairment is a common complication and comorbidity of diabetes. However, the underlying mechanisms of diabetes-associated cognitive dysfunction are currently unclear. M1 microglia secretes pro-inflammatory factors and can be marked by CD16, iNOS, Iba1 and TNF-ɑ. The decline of M2 microglia in the diabetic rats indicates that high glucose promotes the differentiation of microglia into the M1 type to trigger neuroinflammatory responses. Moreover, there is a lack of strong evidence for treatments of diabetes-associated cognitive impairment in addition to controlling blood glucose.

**Methods:** Diabetic rats were established by intraperitoneal injection of one dose of streptozotocin (60 mg/kg). Polarization transitions of microglia were induced by high glucose treatment in BV2 cells. Levetiracetam was orally administered to rats 72 h after streptozotocin injection for 12 weeks.

**Results:** In STZ-induced diabetic rats, the results demonstrated that levetiracetam improved rat cognitive function (*Morris water maze test*) and hippocampus morphology (*Hematoxylin-eosin staining*), and the effect was more evident in the high-dose levetiracetam group. Microglia activation in the hippocampus was inhibited by levetiracetam treatment for 12 weeks. Serum levels of TNF-α, IL-1β, and IL-6 were reduced in the LEV-L and LEV-H groups, and IL-1β level was obviously reduced in the LEV-H group. *In vitro,* we found that levetiracetam 50 µM attenuated high-glucose induced microglial polarization by increasing IL-10 level and decreasing IL-1β and TNF-α levels. Moreover, levetiracetam 50 µM increased and decreased the proportion of CD206+/Iba1+ and iNOS+/Iba1+cells, respectively. Western blot analysis illustrated that LEV 50 µM downregulated the expression of MyD88 and TRAF6, and phosphorylation of TAK1, JNK, p38, and NF-κB p65. The effect of levetiracetam on the anti-polarization and expression of p-JNK and p-NF-κB p65 were partly reversed by anisomycin (p38 and JNK activators).

**Discussion:** Together, our data suggest that levetiracetam attenuates streptozotocin-induced cognitive impairment by suppressing microglia activation. The *in vitro* findings also indicate that the levetiracetam inhibited the polarization of microglia via the JNK/MAPK/NF-κB signaling pathway.

## 1 Introduction

Diabetes is characterized by hyperglycemia due to insulin secretion defects and/or insulin resistance. It might induce many complications and comorbidities, including cognitive dysfunction (McCrimmon et al., 2012). Epidemio-logical data illustrated that cognitive decrease is more prone to patients with diabetes and involves a process from cognitive decrement (Biessels and Despa, 2018), to cognitive impairment, and even dementia (Ennis et al., 2020; Willmann et al., 2020). However, the etiologies and the underlying mechanisms are not very clearly. Metaflammation refers to a combination of metabolic disorders with chronic low-grade inflammation (Hotamisligil, 2017). High levels of activated systemic inflammation factors in DM, such as have been detected in diabetes patients, thus making them potential predictors for cognitive impairment (Marioni et al., 2010; Niewczas et al., 2019; Dyer et al., 2020). Very few clinical studies on the DM related cerebral inflammation have been conducted; however, diabetic animal studies illustrated that cognitive impairment and neuronal injury were associated with the microglia activation (Liu Y. et al., 2018). Natunen et al. have found that in mice with genetic Alzheimer’s disease (AD) and type 2 DM, the scale of microglia near β-amyloid plaques is reduced owing to phagocytosis (Natunen et al., 2020).

Microglia in neurovascular units participate in brain injury response, increase of blood-brain barrier permeability (Prince et al., 2013), synaptic recombination, post-synaptic spinous process formation, nerve regeneration and early connection of the nerve loop. Microglia are categorized into pro-inflammatory (M1) and anti-inflammatory (M2) (de la Monte et al., 2006), mutually transforming into each other in different biological processes (Craft, 2009; Prince et al., 2013; Brady et al., 2017; Kadohara et al., 2017). M1 microglia secretes pro-inflammatory factors and can be marked by CD16, iNOS, Iba1 and TNF-α. In addition, according to flow cytometry results, the decline in M2 microglia in the brain tissue of diabetic rats indicates that high glucose promotes the differentiation of microglia into the M1 type to trigger neuroinflammatory responses. Given that inflammation is associated with cognitive impairment, modulating the ratio of M1/M2 types of microglia might cope with cognitive dysfunction in DM. However, more research is needed before realizing this hypothesis.

Synaptic vesicle glycoprotein 2A (SV2A) is a member of the transporter super-family, and a component of the synaptic vesicle membrane. SV2A is essential for the transport of neurotransmitters, and the shuttle and exocytosis of vesicles. Levetiracetam (LEV), the first identified SV2A ligand, has a broad-spectrum anti-epileptic effect. Chronic epilepsy model was used to evaluate the effect of LEV on histological changes, hippocampal neurogenesis and synaptic plasticity in chronic epilepsy. 54 mg/kg LEV (once daily for 2 weeks, intraperitoneal injection) had no effect on the hippocampal neurogenesis but improved the CA1 subfield volume and synaptic transmission in CA3-CA1 areas (Salaka et al., 2022). These findings provide some bases for the further studies on cognition. Differential effects of levetiracetam were observed on the hippocampal CA1 synaptic plasticity and the dentate gyrus in epileptic rats. Kainite-induced epileptic mice were used to illustrate the effect of subchronic (continuous 7 days) and chronic (continuous 35 days) LEV treatments on the hippocampal neurogenesis. Chornic LEV administration could promote the multiplication of progenitor cell neuroblasts and neural stem cells. While subchronic LEV treatment inhibited the multiplication of progenitor cells neuroblasts, but promoted the multiplication of quiescent neural stem cells (Zhang et al., 2020). Moreover, SV2A dysfunction is involved in many types of cognitive disorders, suggesting the potential therapeutic value of LEV (Stienen et al., 2011).Liu et al. conducted system reviews to evaluate the effects of anti-epileptic drugs on epilepsy in people with AD in 2018 and 2023. In 2018, the results showed no significant differences among generalized used anti-epileptic drugs, such as levetiracetam (LEV), lamotrigine (LTG) and phenobarbital (PB). LEV, regardless of its anti-epileptic effect, may also impair mood. Same results were found in system reviews by Liu in 2023 (Liu S. et al., 2018; Liu et al., 2021). However, LEV exhibits neuroprotective properties in rodent TBI (traumatic brain injury) models. Some other mechanisms are also involved in the neuroprotection of LEV in TBI mouse models, such as inhibiting of mitochondiral dysfunction (Gibbs et al., 2006), decreasing neruoinflammation (Kim et al., 2010) and microglia phagocytosis (Itoh et al., 2019). Pavone et al. found that new AEDs gabapentin, lamotrigine, tiagabine, and LEV did not exert toxic effects on primary rat astrocytes at low and high concentrations, proving that these new AEDs can prevent inflammation, neuronal damage, and death upon pathological stimuli, such as TBI and/or epilepsy (Pavone and Cardile, 2003). Recent studies have suggested that LEV prevents acute neuroinflammation by suppressing inflammatory neutrophil (CD11b+ CD45^+^) infiltration after status epilepticus (SE) (Matsuo et al., 2022). In another study, LEV might inhibit astrocyte reactivity by decreasing the number of A2 astrocyte markers (Komori et al., 2022). In addition to SE, few studies were been conducted to analyze diabetic complications. LEV improves diabetic retinal structure organization, alleviates retinal inflammation, and downregulates retinal GLUT1 expression (Mohammad et al., 2019). Erbas et al. demonstrated that LEV suppressed electrophysiological alterations in the sciatic nerves via inhibiting inflammation and fibrosis, indicating its therapeutic effects against diabetic neuropathy (Erbas et al., 2016). However, the action mechanisms of LEV in treating diabetes-associated cognitive dysfunction remain unclear.

The NF-κB and MAPK signaling pathways are involved in M1 activation, and possibly subsequent inflammatory response (Cho et al., 2019). NF-ĸB transcription activates pro-inflammatory factors (Liu Y. et al., 2018; Liu et al., 2019). In addition, c-Jun, JNKs, p38, and extracellular signal-related kinases (ERK) (Tang et al., 2013) are activated through the MAPK pathway to increase the expression of genes associated with inflammation, apoptosis, and differentiation (Thalhamer et al., 2008). These mechanisms have not been proven to induce the effect of LEV on cognitive deficits in STZ-induced diabetic rats and in high glucose induced inflammation in BV2 cells. Therefore, we designed this experiment to verify the effects of LEV on the cognitive function of STZ-induced diabetic rats *in vivo* and the transitions of microglia and the potential regulatory mechanisms *in vitro.*

## 2 Materials and methods

### 2.1 Animals model and group

Adult male Sprague-Dawley (SD) rats (200 ± 10 g) (Kaswin Laboratory Animals Co.,Ltd., China) were housed in a room with a temperature of 22°C ± 2°C and humidity of 55% ± 5% under a 12-h light/dark cycle. All rats could freely drink and take food. One week later, 30 rats were randomly divided into the control and STZ groups. Animals in the control group were treated with sodium citrate buffer (pH 4.5), whereas animals in the STZ groups were subjected to one dose of intraperitoneal STZ (60 mg/kg, S0130, Sigma-Aldrich) dissolved in 100 mM sodium citrate buffer.The blood glucose in the tail vein exceeded 16.7 mmol/L at 72 h after STZ injection was identified as diabetic rats. Among 24 rats, 20 rats were successfully modeled and divided randomly into the STZ group (gavage saline), LEV low-dose group (LEV-L, gavage 10 mg/kg LEV, 12 weeks), and LEV high-dose group (LEV-H, gavage 100 mg/kg LEV, 12 weeks). LEV (UCB Pharma S.A, resuspended in 0.9%.

NaCl saline, 10 mg/kg; 100 mg/kg) and dissolved in the saline as needed. Fasting blood glucose was measured via using a Bayer blood glucometer at 1, 3, 5, 9, and 12 weeks. Weight Blood was measured at 1, 3, 5, 9, and 12 weeks. Fasting period lasted for 12 h, from 6:00 PM to 6:00 AM of the next day. The rats in four groups were sacrificed at 12 weeks (2 rats died during the experiment). Blood was sampled from the inner canthal orbital vein. Hippocampus slices were collected following polyformaldehyde perfusion. All experiments were approved by the animal ethics committee of Wuxi people’s hospital affiliated to Nanjing medical university.

### 2.2 Morris water maze (MWM) test

The MWM test was performed to examine the spatial learning and memory of rats (Tang et al., 2013). The training session lasted for 5 days, and 4 successive trials were conducted each day, with a 2-h interval. The test session was conducted at day 6. One circular pool (160 cm in diameter, 50 cm in height) with a platform in the center of one quadrant was used to train the rats individually at 25°C. In the training session, four successive trails were conducted each day with a 2-h interval and each trail lasted for 90 s. Once the rat arrived at the platform for the first time, the time was recorded. If the rat could not find the platform within 90 s, the researcher could direct the rat to the platform and the trial ended. Test was carried out only once for rats to find the platform within 90 s after the platform was removed in the test session. The process of rats finding the platform was recorded by video tracking equipment, while the data of retention time in the target quadrant and escape latency were processed by an analysis management system (DigBehV-MG; Ji Liang Instruments, China).

### 2.3 Histopathological examination

Rats were perfused with 4% paraformaldehyde, and the brain was removed. Slices containing hippocampus were embedded in paraffin. For morphological evaluation, 5 µm-thick coronal sections of the hippocampus were cut by paraffin slicer for later use. The sections were dewaxed, hydrated with a series of graded alcohol, and stained with hematoxylin and eosin. Morphological changes in CA1, CA3 and DG areas of hippocampus were then observed under a microscope (Olympus, Japan).

### 2.4 Immunohistochemistry

For immunohistochemical staining, brain hippocampus coronal sections were dewaxed, rehydrated, and incubated with antigen retrieval solution for 20 min to expose antigen epitopes. The slices were incubated with primary antibodies in a 4°C refrigerator overnight. Next, the slices were incubated again with antigen retrieval solution for 20 min, and then washed with PBS three times on a shaker, followed by incubation with secondary antibodies at 37°C for 1 h. After that, the slices were treated with DAB solution (ZLI-9018, Zhongshan Jinqiao, China) for 15 min and subsequently rinsed with distilled water to stop color development. Next, hematoxylin was treated for 10 min to counterstain the nucleus. Having been dehydrated, the slices were sealed and observed under a microscope. The IOD of Iba1 positive cells on each image was calculated.

### 2.5 Cell culture and high-glucose BV2 cell model

Microglia cell line BV2 (CC-Y2022, EK-Bioscience Biotechnology Co., Ltd., ShangHai, China) were cultured in LD-DMEM (5 mM glucose in Dulbecco’s Modified Eagle Medium, BL308A; Biosharp) with 10% fetal bovine serum, 100 U/mL penicillin, and 100 pg/mL streptomycin and incubated at 37°C and 5% CO_2_. Glucose at various concentrations (from 25, 50, 75–100 mM) was added to the cells to establish a BV2 cell model of high glucose-induced inflammation as 25 mM concentration of glucose was the upper limit of normal concentration in culture medium.

### 2.6 Application of levetiracetam and chemicals *in vitro*

To assess the effect of LEV, LEV (HY-B0106; MedChemExpress LLC) was added to the culture medium of BV2 cells, followed by incubation for 24 h. LEV was added at various concentrations to the medium to examine the concentration-response of LEV. Anisomycin (an activator of p38 and JNK, HY-18982; MedChemExpress LLC) was added simultaneously for investigation of the role of the MAPK signaling pathway. Anisomycin concentration gradient was also evaluated in our study.

### 2.7 Cell viability analysis and lactate dehydrogenase (LDH) assay

We used a CCK-8 Kit (CK04; Dojindo) to detect the cell viability. BV2 cells were seeded at 5 × 10^4^/mL and cultured in 96-well plate overnight at 5% CO_2_ and 37°C. 10µL CCK-8 solution was added to each well and incubated for 3 h. At last, the absorbance was measured at 450 nm. The LDH kit (A020-2; Nanjing Jiancheng Bioengineering Institute, China) was added to the 96-well plate to test the cell viability in cell-free supernatant. Briefly, cell-free supernatant was added to the 96-well plate when the cells were cultured in the treatment medium. Double distilled water, 0.2 ul/mL pyruvate standard solution, matrix buffer, coenzyme I and samples were added to the corresponding wells, respectively, followed by incubation for 15 min at 37°C. Then, 25 ul of 2,4-dinitrophenylhydrazine was injected into each well, followed by a 15-min incubation again. After that, NaOH solution was added into each well and reacted for 5 min. Finally, the absorbance was measured at 450 nm.

### 2.8 Immunofluorescence staining

BV2 cells were seeded in 24-well plates for immunofluorescence staining after a glass sheet was prepared in each well. BV2 cells were washed with three times with PBS, fixed with 4% paraformaldehyde for 10 min, blocked by 5% bovine serum blocking solution for 2 h, incubated with primary antibodies (Iba1, CD206 and iNOS) at 4°C for one night. The cells were incubated with the corresponding secondary antibodies for 1 h at the second day. Hochest was added to the cells and reacted for 15 min at room temperature. Finally, the images were observed under a laser confocal microscope (Leica, Germany).

### 2.9 Real time -PCR

Trizol reagent was used to extract total RNA from BV2 cells, and a Revert Aid First Strand cDNA Synthesis kit was used to reverse-transcribe RNA into cDNA (R401-01-AA, Vazyme). The IL-1β, IL-10, TNF-α, iNOS and CD206 mRNA levels were detected using the 2× SYBR Green qRCR Mix. The primes for RT-PCR were shown in Table 1. The mRNA levels of genes were analyzed using 2^−ΔΔCT^ method. The expression of all genes was standardized to the expression of GAPDH as the control GAPDH.

**TABLE 1:** The primes for RT-PCR.

Gene name	Forward prime	Reverse prime
IL-1β	AGCATCCAGCTTCAAATC	ATCTCGGAGCCTGTAGTG
IL-10	ACC​TGG​TAG​AAG​TGA​TGC​C	CACCTTGGTCTTGGAGCT
TNF-α	GTG​GAA​CTG​GCA​GAA​GAG​G	ACAGAAGAGCGTGGTGGC
iNOS	GAG​CGA​GTT​GTG​GAT​TGT​C	CCAGGAAGTAGGTGAGGG
CD206	AGGGTGCGGTACAAC	CAGTAGCAGGGATTTCGT

### 2.10 ELISA

Serum insulin, TNF-α, IL-1β and IL-6 levels were analyzed at 12 weeks after treatment via ELISA following the instructions. The blood samples were detected by (insulin) EILISA kit (DL-INS-Ra; DLdevelop), (IL-1β) ELISA Kit (DL-IL1b-Ra; DLdevelop), (IL-6) ELISA Kit (DL-IL6-Ra; DLdevelop) and (TNF-α) ELISA Kit (DL-TNFα-Ra; DLdevelop). BV-2 cells were seeded at 5 × 10^4^/mL and stimulated with glucose (50 mM) in 96-well plate and then treated with LEV, LEV combined with anisomycin. The medium concentrations of IL-1β, TNF-α, and IL-10 were detected using ELISA kits (E-EL-M0037c, E-EL-M0046c, E-MSEL-M0002, respectively; Elabscience). The absorbance value of the mixture was measured at 450 nm.

### 2.11 Western blotting analysis

Total protein was extracted from BV2 cells, and the concentration was measured using a BCA kit. Equal amount of protein was loaded on a 10% SDS gel, separated by SDS-PAGE, and transferred to PVDF membranes, which was blocked in 5% skim milk for 1 h at room temperature. The membranes were incubated at 4°C overnight with primary antibodies, and then incubated with secondary antibodies at 37°C for 40 min to 1 h. The primary antibodies included pJNK (ab124956, Abcam) and JNK (ab179461, Abcam), p-p38 (ab4822, Abcam), p38 (14064-1-AP, Proteintech), p-p65 (ab76302, Abcam), p65 (ab16502, Abcam), MyD88 (4283S, CST), TRAF6 (ab33915, Abcam), TAK1(4505S, CST), p-TAK1(9339S, CST). For detection of the MyD88 and TRAF6 proteins, individual gel was run for each immunoblotted protein, with β-actin as an endogenous control. The membranes were stripped by stripping buffer (P0025, Beyotime) after probing MyD88 or TRAF6, and then incubated with the primary and the corresponding secondary antibodies of β-actin, respectively. To detect the expression of phosphorylated protein and the corresponding total protein, such as pJNK and JNK, one gel was run for each protein, with β-actin used as an endogenous control. The same method was applied for measuring the levels of p-p38 and p38, p-p65 and p65, and TAK1 and p-TAK1 expression. After probing for phosphorylated protein, the membranes were stripped using stripping buffer and then incubated with the primary and the secondary antibodies against the corresponding total protein. At last, the membranes were stripped as described above and incubated with the primary and the corresponding secondary antibodies against β-actin. The protein level was normalized into that in the control group. The bands were analyzed using the ImageJ software.

### 2.12 Statistical analysis

The SPSS 20.0 software was introduced for analysis. Measurement data were expressed as the mean ± standard deviation. One way ANOVA was carried out for comparison between groups. LSD (L) or Dunnett’s test was conducted for inter-group analysis. Two-way repeated measures analysis of variance was used to evaluate the role of time and different treatment for blood glucose and body weight. GraphPad Prism 8 was used to construct a graph for the results of data analysis. Differences were considered to be statistically significant when *p* < 0.05.

## 3 Results

### 3.1 LEV ameliorated cognitive deficit and hippocampal injury in STZ-induced rats at 12 weeks

**FIGURE 1:**
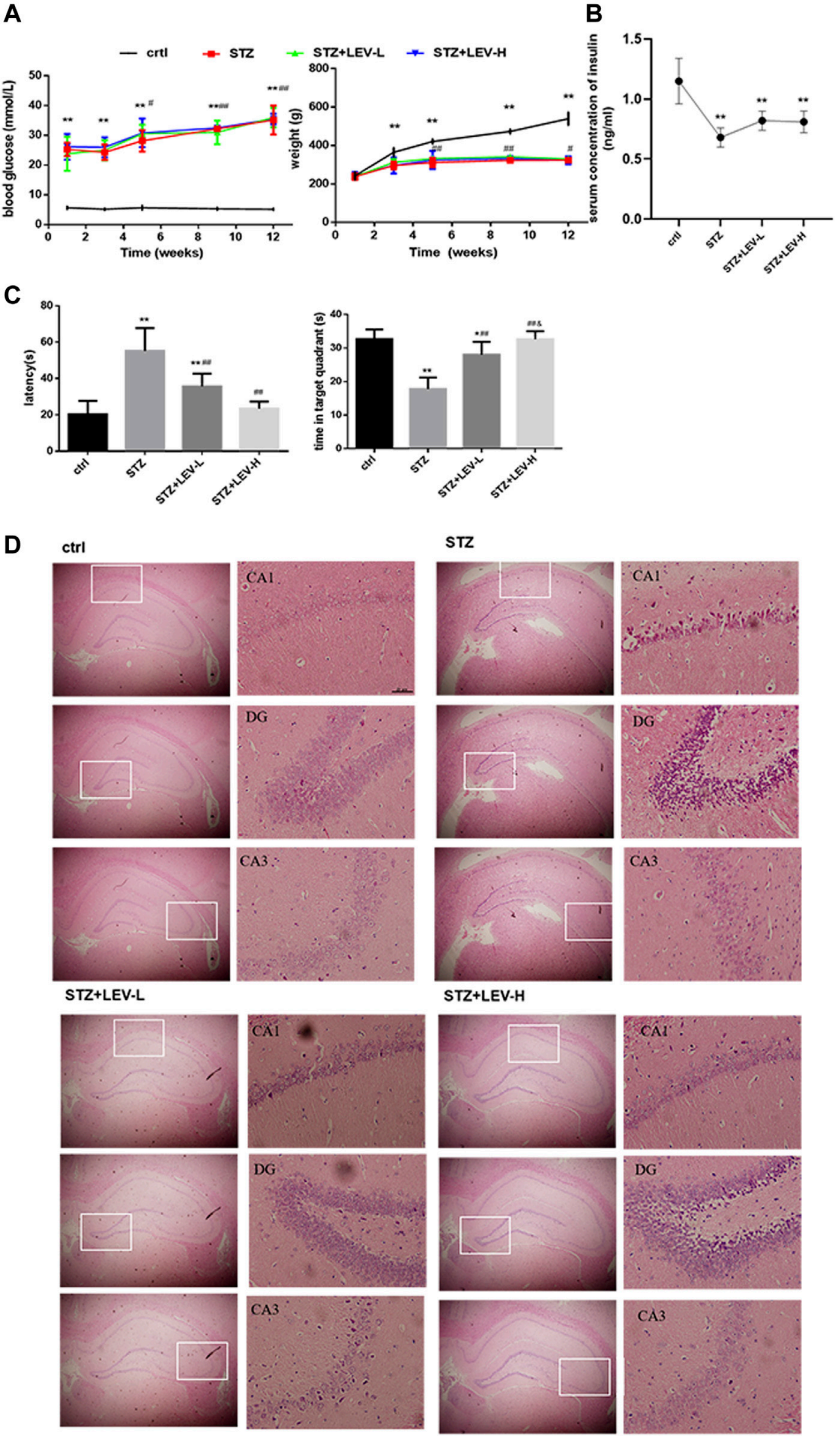
LEV ameliorated cognitive deficit and the injury in the hippocampus of STZ-induced rats at 12 weeks **(A)** Blood glucose levels and body weights at weeks 1, 3, 5, 9, 12 of STZ-induced rats treated with low-dose and high-dose LEV or saline. An interaction between time and different treatments (STZ and STZ + LEV) was analyzed via two-way repeated measures analysis of variance. No interaction between group and time for blood glucose and weight (F time × group = 0.420, *p* = 0.905 df 8; F time × group = 0.474, *p* = 0.870 df 8). Blood glucose level in experimental groups increased at 1,3,5,9 and 12 weeks after the beginning of the experiment (F time = 30.436, *p* = 0.000, df 4). One single dose of STZ continuously increased the blood glucose level at weeks 1(F = 39.78, *p* = 0.000), 3 (F = 77.03, *p* = 0.000), 5 (F = 76.54, *p* = 0.000), 9 (F = 270.501, *p* = 0.000) and 12 (F = 145.62, *p* = 0.000). The blood glucose levels at weeks 5,9 and 12 were significantly increased compared with those at weeks 1 (*p* =0.023, *p* =0.001, *p* =0.000), but without significant difference among three expeimental groups (F group = 0.914, *p* = 0.422, df 2). The body weight decreased at weeks 3 (F = 8.759, *p* = 0.001), 5 (F = 19.89, *p* = 0.000), 9 (F = 198.53, *p* = 0.000) and 12 (F = 153.00, *p* = 0.000) after STZ treatment.The weights at weeks 5, 9, 12 were higher than those at week 3 (*p* = 0.003, *p* = 0.000, *p* = 0.044), but without significant difference among three expeimental groups (F group = 0.975, *p* = 0.400 df 2). **(B)** The serum concentration of insulin of STZ-induced diabetic rats in four groups at week 12. A single dose of STZ also decreased the serum concentration of insulin (F = 15.97, *p* = 0.000 df 3). Compared with control group,*p < 0.05, **p < 0.01; Compared with week 1 or week 3, ^#^ *p* < 0.05, ^##^ *p* < 0.01 Fig **(A,B)**. **(C)** Escape latency and time in target quadrant were recorded at day 6 in four groups. The EL was prolonged and the target quadrant residence time was shortened after STZ treatment (F = 21.37, *p* = 0.000 df 3) Compared with control group,*p < 0.05, **p < 0.01; Compared with STZ group, ^#^ *p* < 0.05, ^##^ *p* < 0.01; Compared with LEV-L group, ^&^ *p* < 0.05). **(D)** H&E staining of hippocampus from control and STZ-induced rats treated with low-dose and high-dose LEV at week 12. LEV-L, 10 mg/kg; LEV-H, 100 mg/kg. Data are expressed as mean ± SEM. n = 6/group.

To verify the successful establishment of the diabetic rat model, blood glucose level and body weight were tested at weeks 1, 3, 5, 9 and 12, while the insulin level at week 12. An interaction between time and different treatments (STZ and STZ + LEV) was searched in our study. Two-way repeated measures analysis of variance showed that there was no interaction between group and time for blood glucose and weight (F time × group = 0.420, *p* = 0.905; F time × group = 0.474, *p* = 0.870). Blood glucose level in experimental groups increased at 1,3,5,9 and 12 weeks after the beginning of the experiment (F time = 30.436, *p* = 0.000). One single dose of STZ continuously increased the blood glucose level at weeks 1, 3, 5, 9 and 12 (*p* = 0.000 for all). The blood glucose levels at weeks 5, 9 and 12 were significantly increased compared with those at weeks 1 (*p* = 0.023, *p* = 0.001, *p* = 0.000), but without significant difference among three expeimental groups (F group = 0.914, *p* = 0.422). The body weight decreased at weeks 3, (*p* = 0.001) 5, 9 and 12 after STZ administration (*p* = 0.000 for all). The weights at weeks 5, 9, 12 were higher than those at week 3 (*p* = 0.003, *p* = 0.000, *p* = 0.044), but without significant difference among three expeimental groups (F group = 0.975, *p* = 0.400). In conclusion, LEV had no effect on the blood glucose and body weight of rats. A single dose of STZ also decreased the serum concentration of insulin (F = 15.97, *p* = 0.000) and no difference was found between the three experimental groups (*p* = 0.059, *p* = 0.094, *p* = 0.814). The above results indicated that the diabetic rat model was successfully established (Figures 1A,B). As shown in Figure 1C, the escape latency (EL) was prolonged and the target quadrant residence time was shortened after STZ treatment (F = 21.37, *p* = 0.000). Low and high doses of LEV could reduce the escape latency (*p* = 0.001; *p* = 0.000) and prolong target quadrant residence time after STZ treatment (*p* = 0.000; *p* = 0.000). The LEV-H group showed a greater improvement in the target quadrant residence time (*p* = 0.02).

As shown in Figure 1D, the experimental groups had looser pyramidal cells in the CA1 region and larger nuclei than the control group, but these changes were alleviated in the LEV-L and LEV-H groups. The LEV-L group exhibited thickened cell layer and increased number of neuronal cells in the DG region. These changes were more evident in the LEV-H group. The cells in the CA3 region were neatly arranged after treatment with LEV. Furthermore, the pyramidal cell layer was much thicker in the LEV-H group than in the other groups.

### 3.2 LEV treatment reduced the excessive activation of microglia in the hippocampus of STZ-induced rats at 12 weeks

**FIGURE 2:**
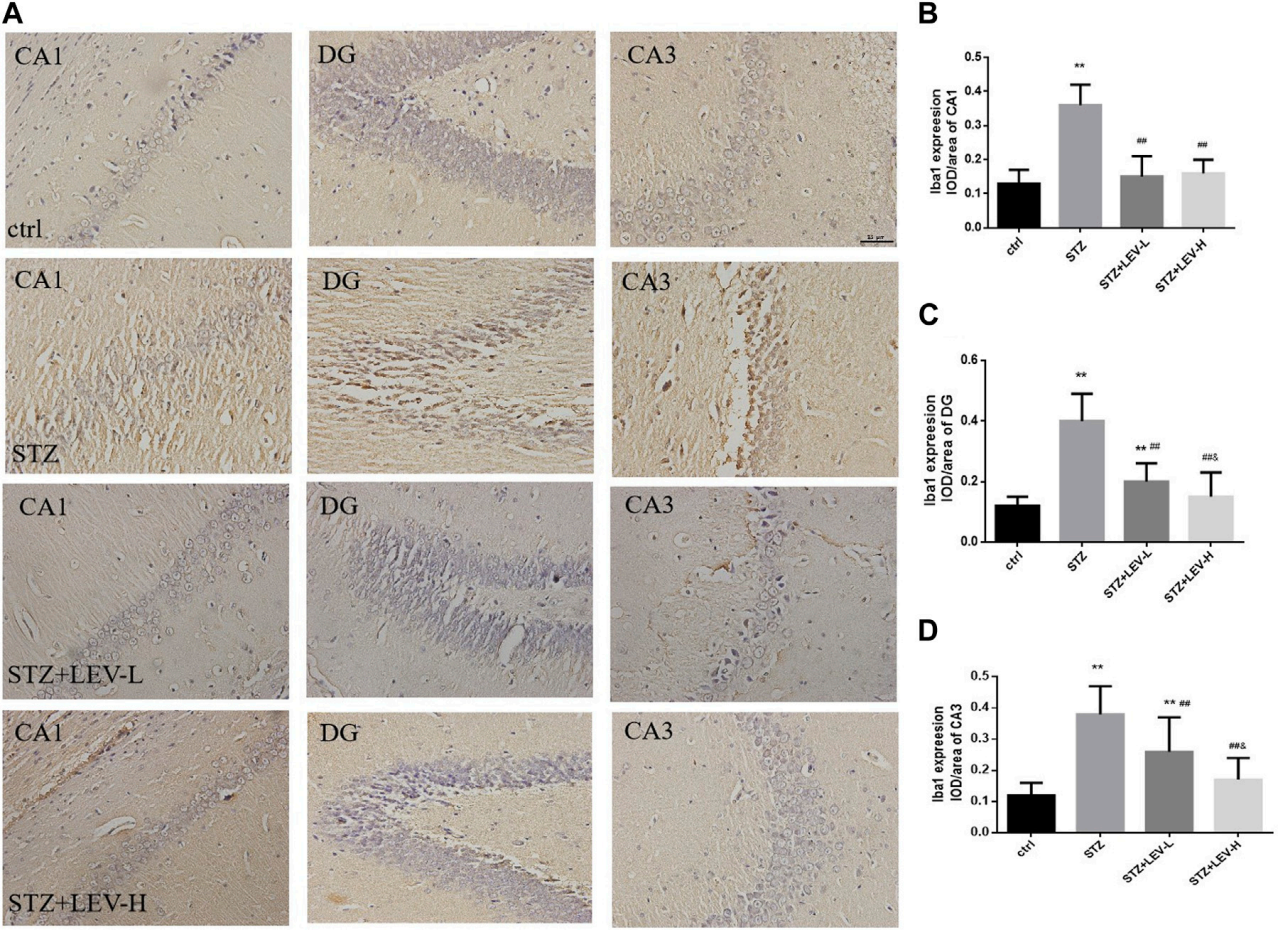
**(A–D)** Levetiracetam decreased the expression of Iba1 in the hippocampus of STZ-induced diabetic rats **(A)** Immunohistochemical staining of the protein expressions of Iba1 in hippocampus from control and STZ rats treated with LEV on week 12. **(B–D)** Analysis of expressions of Iba1 in CA1, DG and CA3 regions in hippocampus of four groups (n = 6/group. One-way ANOVA was used to data analysis (df 3). Compared with control group, *p < 0.05; **p < 0.01; Compared with STZ group, ^#^ *p* < 0.05, ^##^ *p* < 0.01; Compared with LEV-L group, ^&^ *p* < 0.05, ^&&^ *p* < 0.01).

As shown in Figures 2A–D, the proportions of Iba1+ cells in the CA1(F = 39.34, *p* = 0.000), CA3 (F = 153.92, *p* = 0.000) and DG (F = 147.82, *p* = 0.000) regions of the hippocampus were raised after STZ treatment. LEV treatment could decrease the proportion of Iba1+ cells in the CA1, CA3 and DG regions after STZ treatment (*p* = 0.000 for all). Furthermore, the LEV-H group showed a greater decrease in the expression of Iba1 in the DG (*p* = 0.01) and CA3 (*p* = 0.01) regions than the LEV-L group.

### 3.3 LEV decreased serum levels of inflammatory factors in STZ-induced rats at 12 weeks

**FIGURE 3:**
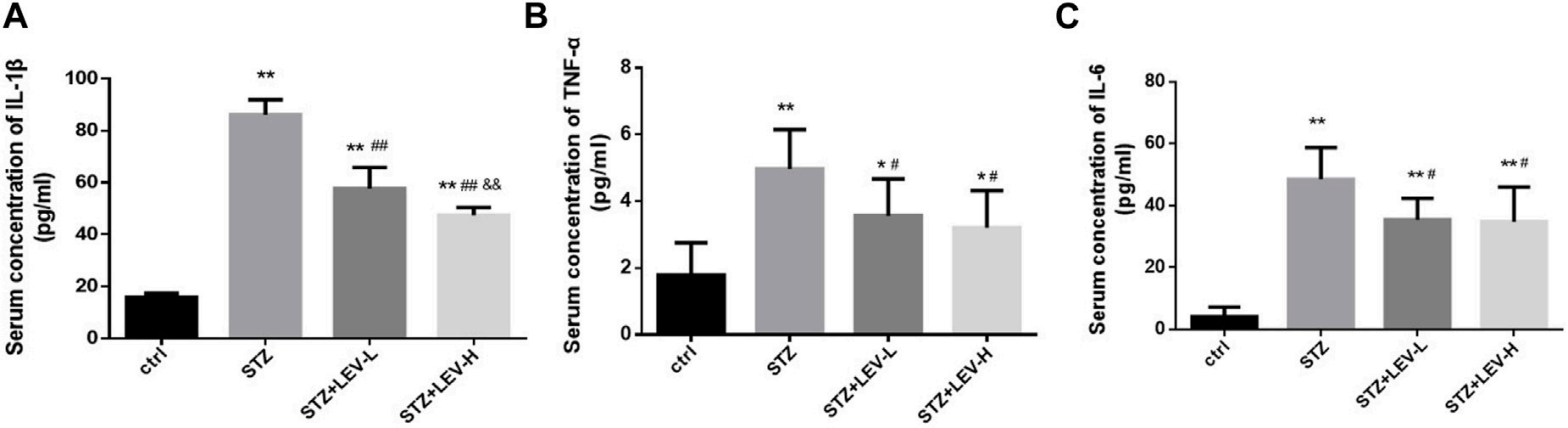
LEV reduced the serum concentration of IL-1β, TNF-α and IL-6 of STZ-induced diabetic rats in four groups **(A–C)** Analysis of serum levels of IL-1β, TNF-α and IL-6 in control group, STZ induced-diabetic rats and LEV-L and LEV-H groups at 12 weeks after STZ Intraperitoneal injection. (n = 6/group. One-way ANOVA was used for data analysis (df 3). Compared with control group, *p < 0.05; **p < 0.01; Compared with STZ group, ^#^ *p* < 0.05, ^##^ *p* < 0.05; Compared with LEV-L group, ^&^ *p* < 0.05, ^&&^ *p* < 0.01).

Serum IL-1β (F = 181.73, *p* = 0.000), IL-6 (F = 29.54, *p* = 0.000) and TNF-α (F = 8.6, *p* = 0.001) levels were also higher in the three experimental groups. Low and high dose of LEV treatment could reduce the serum levels of IL-1β (*p* = 0.000, *p* = 0.000), IL-6 (*p* = 0.015, *p* = 0.012) and TNF-α (*p* = 0.038, *p* = 0.012). Furthermore, the group administered with high-dose LEV exhibited a greater decrease in the serum IL-1β level (*p* = 0.003) (Figures 3A–C).

### 3.4 High glucose induced the activation and polarization of BV2 cells

**FIGURE 4:**
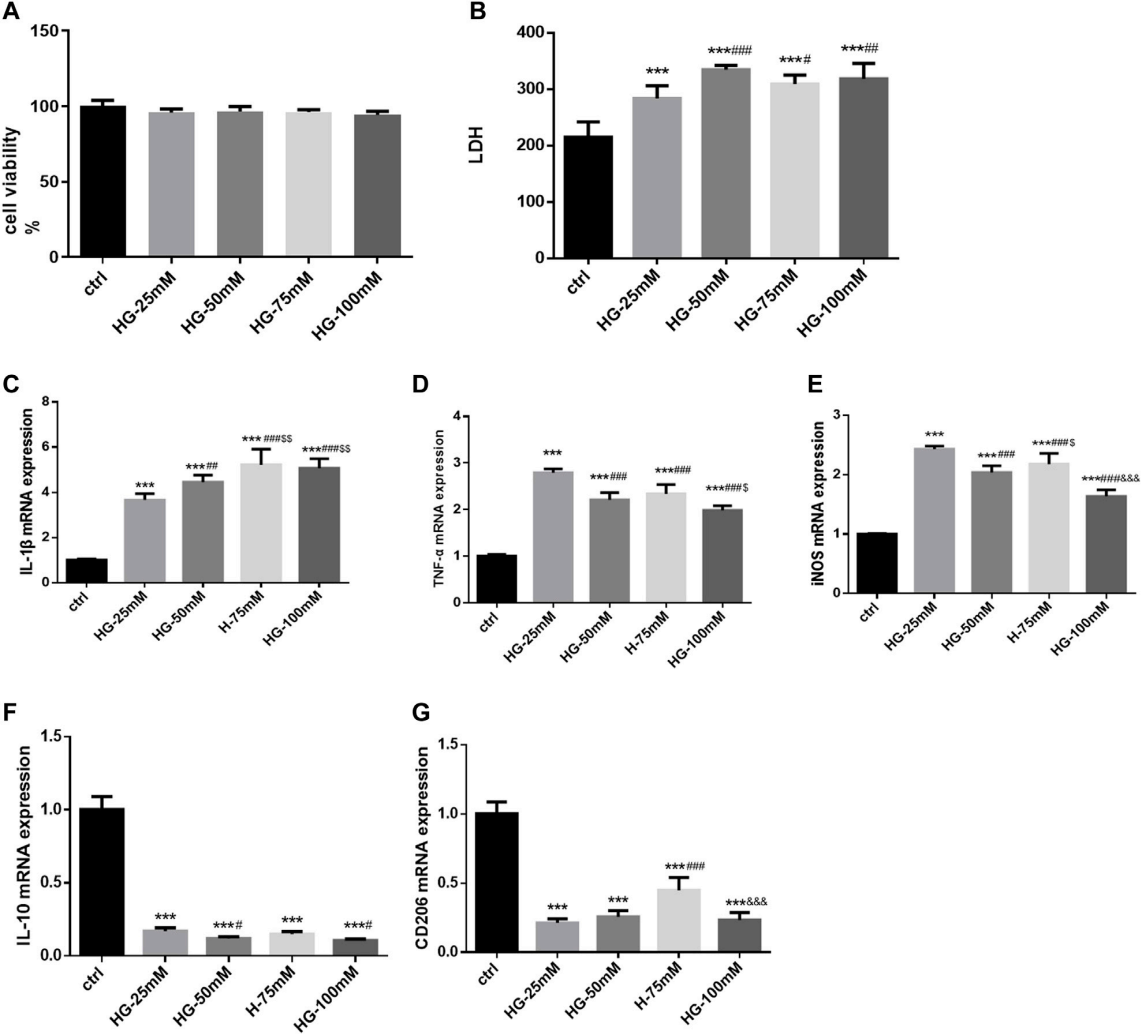
The cell viability, LDH release, activation and polarization of BV2 cells after high-glucose stimulation **(A)** The cell viability of BV2 cells in control and 25 mM, 50 mM, 75 mM and 100 mM glucose treatment groups for 24 h. **(B)** The LDH release of BV2 cells in control and various glucose concentration stimulated groups for 24 h. **(C–G)** The mRNA levels of IL-1β, TNF-α, iNOS, CD206 and IL-10 were measured in BV2 cells by RT-PCR assay and normalized to that of β-actin. Data are expressed as mean ± SEM, n = 6/group (One-way ANOVA was used to data analysis df 4) (Compared with control group, *p < 0.05, **p < 0.01, ***p < 0.001; Compared with 25 mM group, ^#^ *p* < 0.05, ^##^ *p* < 0.01, ^*###*^ *p <* 0.001; Compared with 50 mM group group, ^$^ *p* < 0.05; Compared with 75 mM group, ^&^ *p* < 0.05, ^&&^ *p* < 0.01,^&&&^ *p* < 0.001).

Following high glucose stimulation for 24 h, cell viability did not change significantly (F = 1.595, *p* = 0.250), as shown by the CCK-8 assay (Figure 4A). LDH release was higher after treatment with high glucose at various concentrations (25, 50, 75, and 100 mM). LDH release was more higher in the 50, 75, and 100 mM groups (F = 12.09,*p =*0.001), without a significant difference among the three glucose concentration groups (Figure 4B). As Iba1 was the marker of microglia, the expression of it was just used to identify BV2 cells. Detecting the expression of Iba1 alone could not illustrate the activation of BV2 cells. Could we remove the Immunofluorescence staining results from our study. High glucose markedly increased the expression of inflammatory genes at the mRNA level in BV2 cells, such as IL-1β (F = 40.98), TNF-α (F = 66.42), and iNOS (F = 66.42) (*p* = 0.000 for all). The mRNA level of IL-1β was higher in the 50, 75, and 100 mM glucose groups, than in 25 mM group; while it was much higher in the 75 and 100 mM glucose groups than in 50 mM group. Furthermore, 25 mM glucose increased the expression of TNF-α and iNOS mRNA more prominently than 50, 75, and 100 mM glucose. In TNF-α mRNA expression, 50 mM and 75 mM glucose brought no significantly differences. iNOS mRNA expression was higher in 75 mM glucose group than in 50 mM group. In addition, high glucose treatment inhibited the mRNA expression of IL-10 (F = 208.9, *p* = 0.000) and CD206 (F = 62.12, *p* = 0.000). Compared with the 25 mM group, the 50 and 100 mM groups exhibited decreased IL-10 mRNA expression (all *p* < 0.05). CD206 mRNA expression in the 25 and 50 mM groups was lower than that in the 75 mM group, but without significant difference between the 25 and 50 mM groups. Considering its effects on cell morphology, LDH release, and cell viability, 50 mM was determined as the optimum glucose concentration to establish high glucose-induced inflammation model in BV2 cells. CD206 and iNOS are markers of M2 and M1 microglia, and our results showed that the high glucose treatment induced inflammation and polarization of BV2 cells from M2 to M1 (Figures 4C–G).

### 3.5 LEV attenuated high glucose-induced inflammation in BV2 cells by regulating cell polarization

**FIGURE 5:**
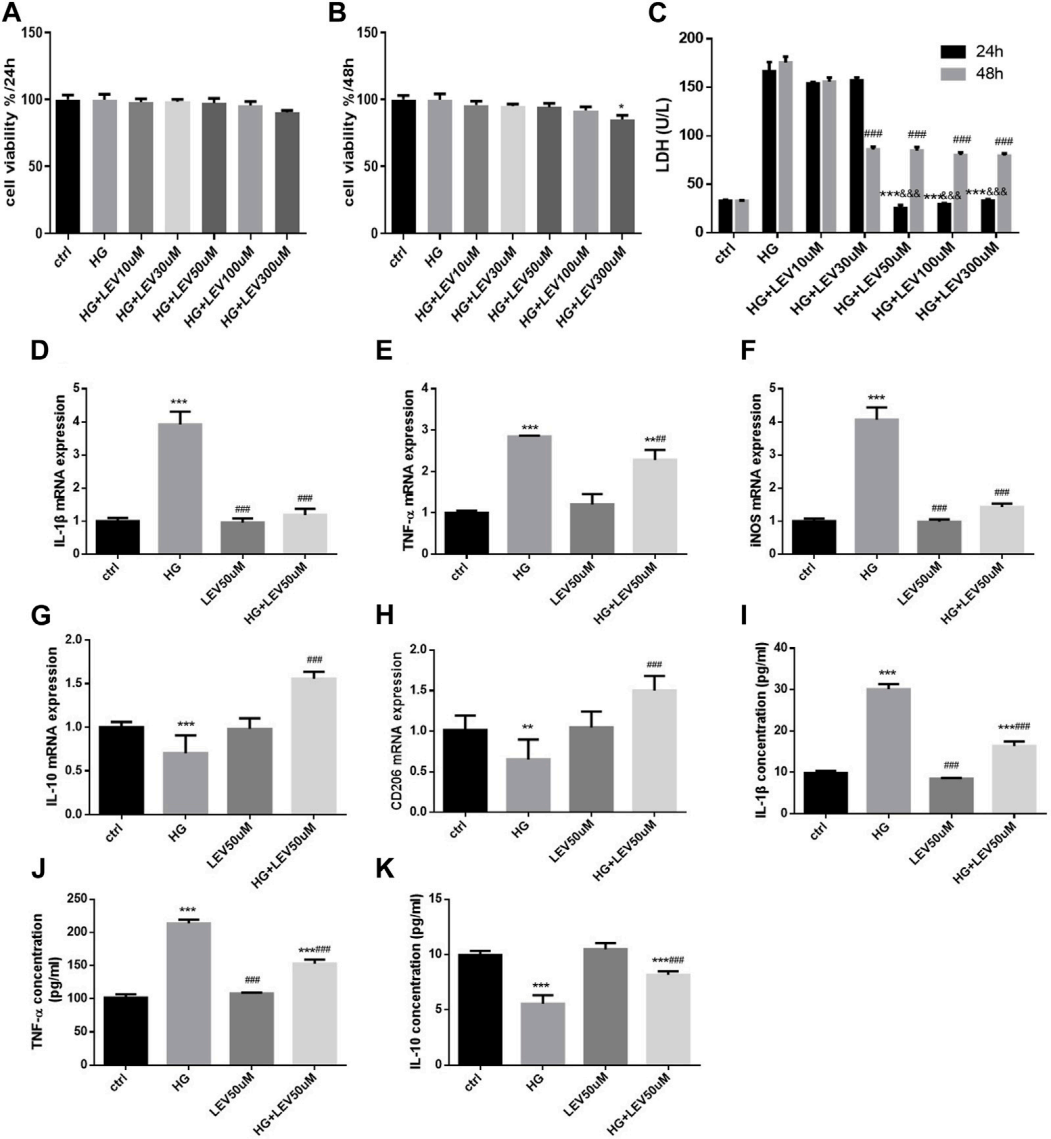
LEV attenuates high glucose-induced cell viability decline, LDH release and microglia polarization **(A–B)** BV2 cells viability detection after various concentration of LEV treatment for 24 h and 48 h. **(C)** The LDH release of BV2 cells after treatment with different concentration of LEV for 24 h and 48 h (Values are expressed as the mean ± SD, n = 6; ^***^ *p* < 0.001 versus the HG group for 24 h, ^###^ *p* < 0.001 versus the HG for 48 h, ^&&&^ *p* < 0.001 compared the LDH release at 24 h with the other at 48 h in each group). **(D–F)** The mRNA expressions of IL-1β, TNF-α and iNOS in BV-2 cells were tested by RT-PCR after treated with 50 uM LEV for 24 h **(G–H)** The mRNA expressions of IL-10 and CD206 in BV-2 cells after treated with 50 uM LEV for 24 h were detected via RT-PCR. **(I–K)** The inflammatory factor levels in supernatant of BV-2 cells after treatment with 50 uM LEV for 24 h were checked via ELISA. Data are expressed as mean ± SEM, n = 6/group (One-way ANOVA was used to data analysis df 6) (Compared with control group, ^*^ *p* < 0.05, ^**^ *p* < 0.01, ^***^ *p* < 0.001; compared with HG group, ^#^ *p* < 0.05, ^##^ *p* < 0.01, ^###^ *p* < 0.001).

As shown in Figures 5A,B, different concentration of LEV treatment for 24 h resulted in no decrease in cell viability in BV2 cells with 50 mM glucose induced-inflammation (F = 3.955, *p* =0.118). 300 μM LEV treatment for 48 h decreased the cell viability compared to the control (*p* =0.037). Treatment with LEV for 24 h reduced LDH release in the 50, 100, and 300 µM groups, with no significant difference between these groups. Treatment with LEV for 48 h reduced LDH release in the 30, 50, 100, and 300 µM groups, with no significant difference between these groups. However, treatment with 50, 100, and 300 µM LEV caused a greater decrease in LDH release at 24 h than that at 48 h (all *p* < 0.001, Figure 5C). The findings suggested that 50 µM LEV was the optimal concentration for treatment. LEV of 50 µM decreased the IL-1β, TNF-α, and iNOS mRNA expressions after high glucose stimulation in BV2 cells (*p* =0.000; *p* =0.004 and *p* =0.000 Figures 5D–F). Moreover, the mRNA expression of IL-10 and CD206 was upregulated after LEV 50 µM treatment (*p* =0.000 for all, Figures 5G,H). The levels of inflammatory factors in the supernatant of BV-2 cells after treatment with 50 µM LEV for 24 h were detected by ELISA. LEV of 50 µM decreased the levels of IL-1β and TNF-α (*p* = 0.000 for all)*,* but increased the level of IL-10 in BV2 cells after high glucose-induced inflammation and polarization (*p =*0.000) (Figures 5I–K).

### 3.6 Anisomycin weakened the anti-inflammatory and anti-polarization effects of LEV in high glucose-stimulated BV-2 cells

**FIGURE 6:**
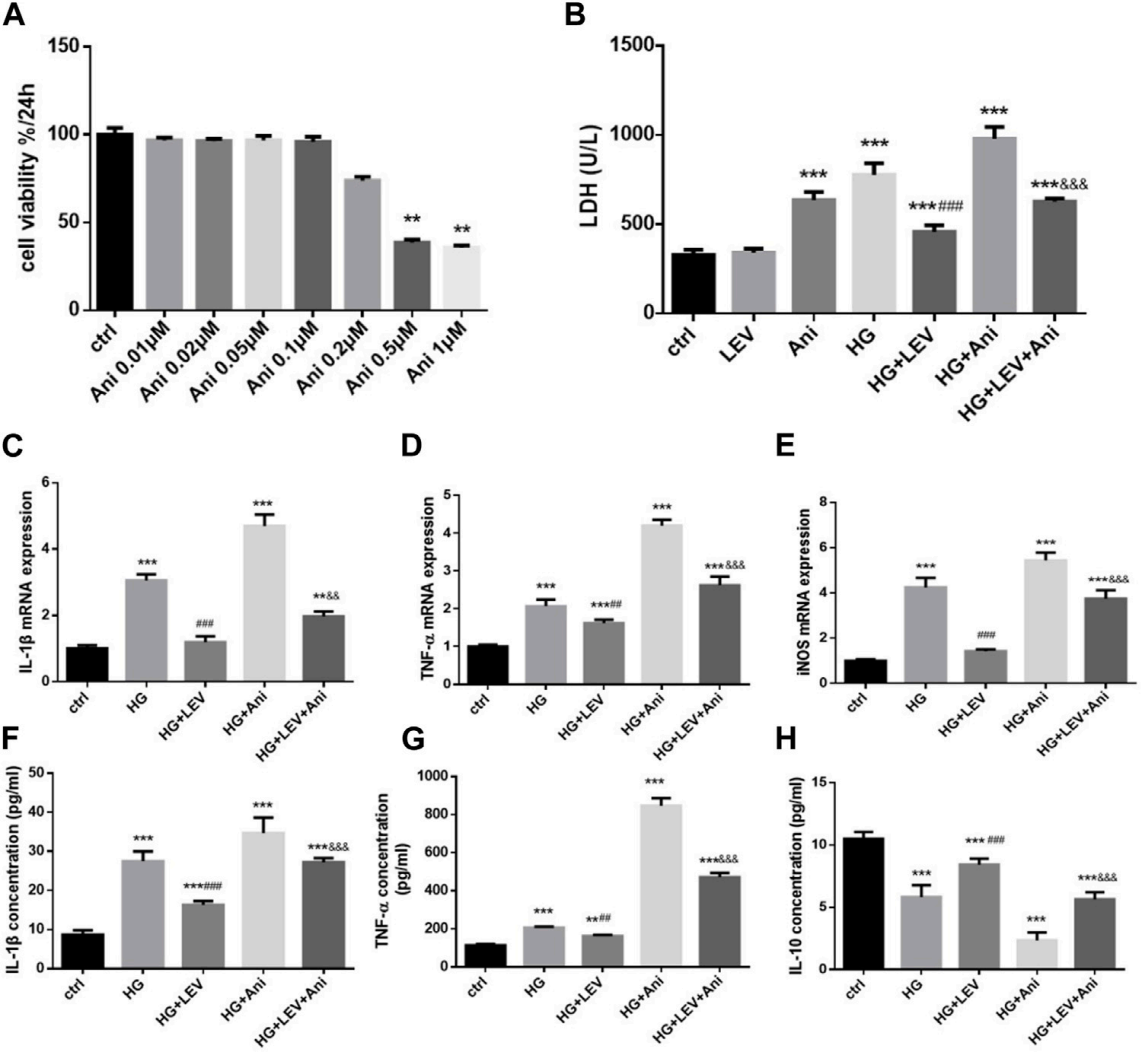
Anti-polarization effects of LEV were blocked by Anisomycin in BV-2 cells induced by high glucose **(A)** BV2 cells viability detection after various concentration of Anisomycin treatment for 24 h. **(B)** The LDH release of BV2 cells in high glucose stimulated after treated with LEV plus Anisomycin (Values are expressed as the mean ± SD, n = 6; ^***^ *p* < 0.001 versus the HG group for 24 h, ^###^ *p* < 0.001 versus the HG for 48 h, ^&&&^ *p* < 0.001 compared the LDH release at 24 h with the other at 48 h in each group). **(C–E)** The mRNA expressions of pro-inflammatory genes in 50 mM glucose stimulated BV-2 cells detected by RT-PCR after treated with LEV plus Anisomycin. **(F–H)** IL-1β, TNF-α and IL-10 factors in BV-2 cells supernatant were tested by ELISA in five groups. Values are expressed as the mean ± SD. n = 6 (One-way ANOVA was used to data analysis df 4) (Compared with control group, *p < 0.05, **p < 0.01, ***p < 0.001; compared with HG group, ^#^ *p* < 0.05,^##^ *p* < 0.01, ^*###*^ *p <* 0.001; compared with HG + LEV group, ^&^ *p* < 0.05, ^&&^ *p* < 0.01,^&&&^ *p* < 0.001).

**FIGURE 7:**
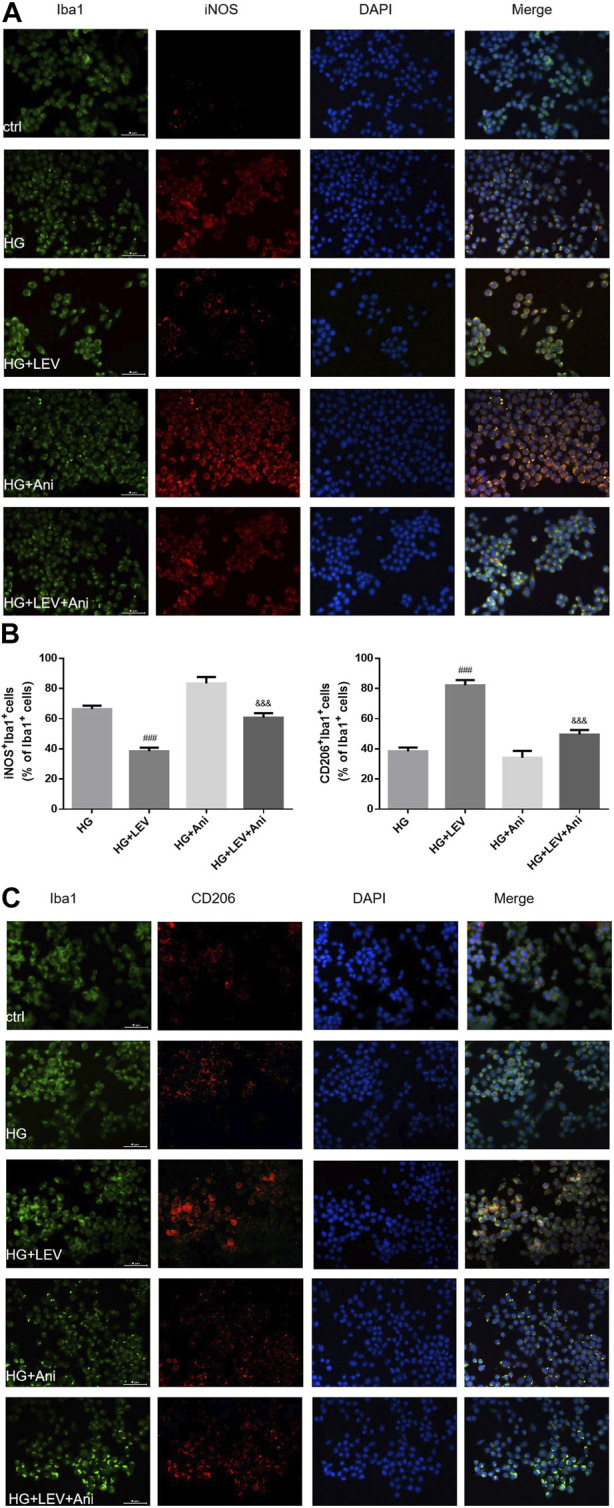
The Iba1/CD206 and iNOS/CD206 co-localization expression in high glucose induced BV2 cells in five groups by immunofluorescence **(A–C)** The proportion of iNOS^+^/Iba1^+^ cells was decreased after LEV 50 uM treatment, while that of CD206^+^/Iba1^+^ was increased. The anti-polarization role of LEV weakens via Anisomycin treatment for 24 h in high glucose induced BV2 cells. Values are expressed as the mean ± SD. n = 6 (One-way ANOVA was used to data analysis df 3) (Compared with HG group, ^*###*^ *p <* 0.001; compared with HG + LEV group, ^&&&^ *p* < 0.001).

The cell viability declined after administration with 0.5 and 1 µM anisomycin for 24 h, as measured by CCK-8 assay. Therefore, we used anisomycin concentration of 0.2 µM in our study (Figure 6A). LDH release was inhibited by 50 µM LEV, but the effect was weakened by 0.2 µM anisomycin, as shown by the LDH release assay results (F = 168.4, *p* = 0.000; Figure 6B). High glucose treatment markedly increased inflammatory genes mRNA expressions in BV2 cells, such as IL-1β (F = 40.98, *p* = 0.000), TNF-α (F = 66.42, *p* = 0.000), and iNOS (F = 66.42, *p* = 0.000). LEV 50 µM decreased IL-1β, TNF-α, and iNOS mRNA expressions after 50 mM high glucose stimulation in BV2 cells (*p =* 0.000; *p* = 0.005 and *p* = 0.000). As a result, IL-1β, TNF-α, and iNOS mRNA expressions were slightly increased in the HG + LEV + Ani group than in the HG + LEV group (*p =* 0.007; *p* = 0.000; *p* = 0.000 Figures 6C–E). LEV of 50 uM decreased IL-1β and TNF-α levels in BV2 cell supernatant (*p =* 0.000; *p =* 0.008), but this effect was partly reversed by anisomycin (*p =* 0.000 for all). IL-10 level in BV2 cell supernatant was increased in the 50 µM LEV group (*p =* 0.000) but decreased in the anisomycin combined with LEV group (*p =* 0.000). These results indicated that anisomycin could weaken the effect of LEV on microglia polarization in BV-2 cells stimulated by high glucose (Figures 6F–H). Immunofluorescence staining was performed to further examine the anti-polarization effect and mechanisms of LEV. Based on Iba1/CD206 and iNOS/CD206 co-localization, iNOS+/CD206+ BV2 cells presented microglial polarization from M1 to M2. After 50 mM high glucose stimulation, LEV 50 µM could decrease the proportion of iNOS + cells and increase that of CD206+ cells (*p* = 0.000 for all). Taken together, these results implied that LEV alleviated high glucose-induced microglial polarization by increasing CD206 expression and decreasing iNOS expression (Figures 7A–C). However, the anti-polarization effect of LEV was partly eliminated by anisomycin (Figures 7A–C).

### 3.7 LEV suppressed microglial polarization via JNK/MAPK/NF-κB in high glucose-stimulated BV-2 cells

**FIGURE 8:**
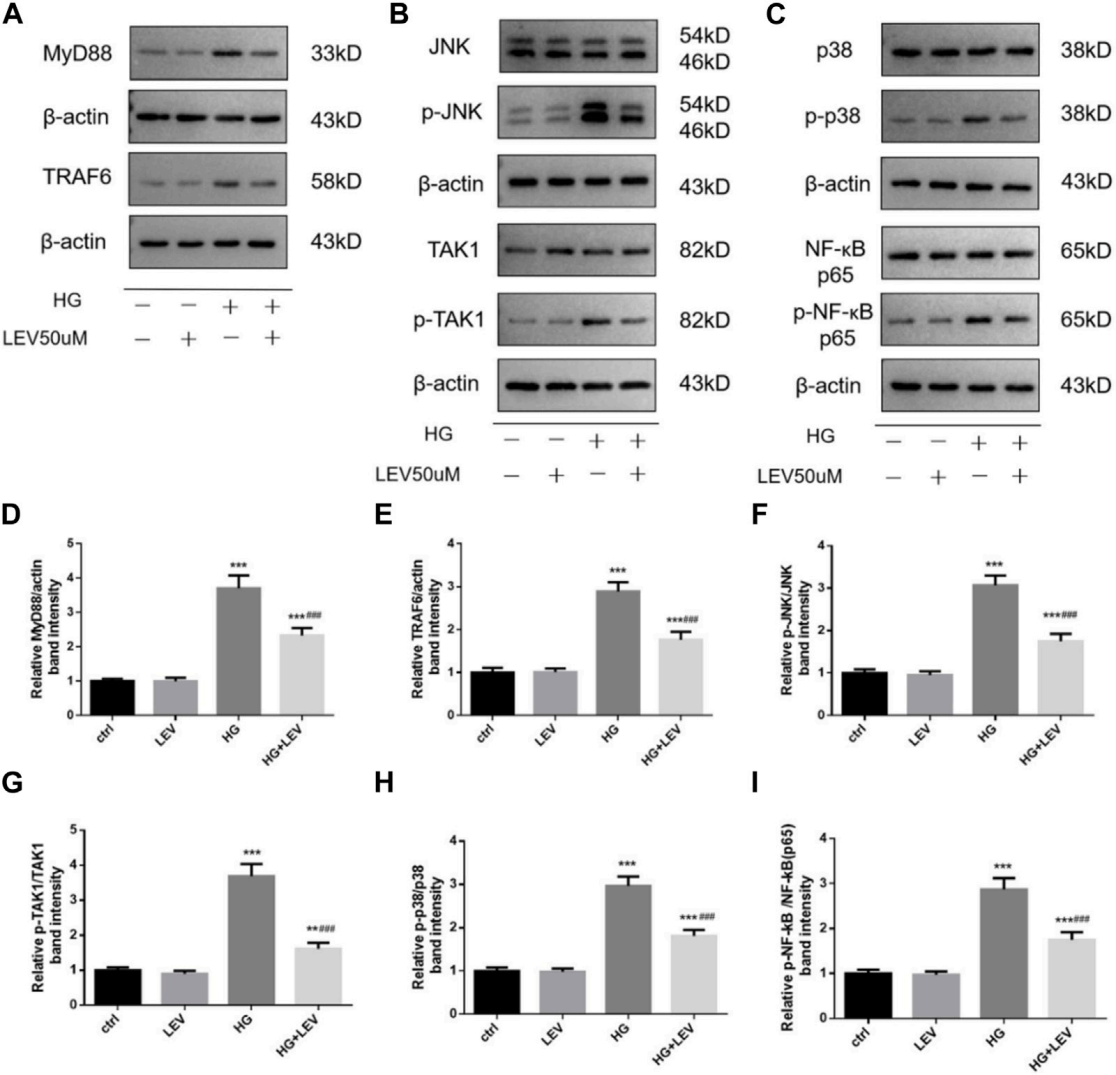
Effect of LEV on the expression of MyD88, TRAF6 and the phosphorylation of TAK1, JNK, p38 and NF-ĸB p65 in BV2 cells. **(A–C)** The expression of MyD88, TRAF6 and the phosphorylation of TAK1, JNK, p38 and NF-ĸB p65 in BV2 cells in four groups **(D–I)** Analysis of the expression of MyD88, TRAF6 and the phosphorylation of TAK1, JNK, p38 and NF-ĸB p65 in ctrl, HG, LEV, HG + LEV groups. Values are expressed as the mean ± SD. n = 5–6 (One-way ANOVA was used to data analysis df 3) (Compared with control group, *p < 0.05, **p < 0.01, ***p < 0.001; compared with HG group, ^*#*^ *p <* 0.05, ^*##*^ *p <* 0.01, ^*###*^ *p <* 0.001).

**FIGURE 9:**
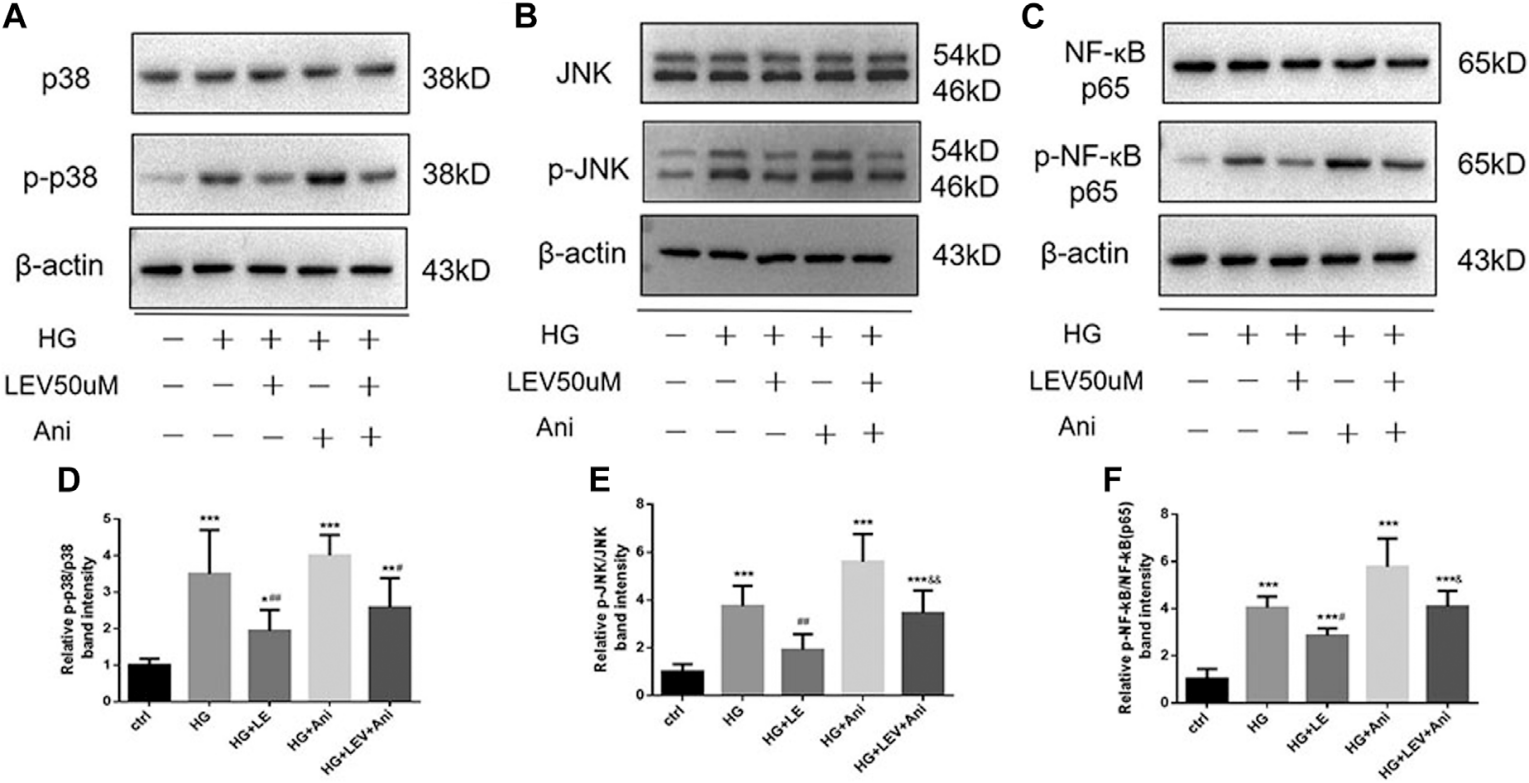
Western blot assay showed the phosphorylation of JNK, p38 and NF-ĸB p65 in BV2 cells treated with LEV and Anisomycin. **(A–C)** The expression of p-p38, p38, p-JNK, JNK, p-NF-ĸB p65 and NF-ĸB p65 in BV2 cells in five groups **(D–F**) Analysis of the ratio of p-p38/p38, p-JNK/JNK, p-NF-ĸB p65/NF-ĸB p65 in ctrl, HG, HG + LEV, HG + Ani and HG + LEV + Ani groups. Values are expressed as the mean ± SD. n = 6 (One-way ANOVA was used to data analysis df 4) (Compared with control group, **p < 0.01, ***p < 0.001; compared with HG group, ^##^ *p* < 0.01, ^*###*^ *p <* 0.001; compared with HG + LEV group, ^&^ *p* < 0.05, ^&&^ *p* < 0.01).

As shown in Figures 8A–I, the expression of MyD88, TRAF6, TAK1, p-TAK1, JNK, p-JNK, p38, p-p38, NF-κB p65, p-NF-κB-p65 was detected in microglia with high glucose-induced polarization after treatment with 50 µM LEV. However, 50 µM LEV decreased the expression of MyD88 and TRAF6, compared to high glucose. The phosphorylation of TAK1, JNK, p38 and NF-κB p65, induced by high glucose, was decreased in the LEV treatment group (*p* = 0.000 for all). These results showed that LEV suppressed high glucose-induced microglial polarization via the MAPK/NF-κB signaling pathway. To further elucidate the anti-polarization mechanisms of LEV, anisomycin was added to BV2 cells simultaneously. The effect of LEV on the phosphorylation of JNK (*p =* 0.001) and NF-κB p65 (*p =* 0.012) was partly reversed by anisomycin. However, no significant difference in the ratio of p-p38/p38 between the HG + LEV and HG + LEV + Ani groups were found in our study (*p =* 0.149 Figures 9A–F). This suggested that the JNK/MAPK/NF-κB signaling pathways were involved in the anti-polarization effect of LEV on high glucose-induced microglial activation and polarization from M2 to M1.

## 4 Discussion

Cognitive impairment is a complication of DM. Our study showed that, at 72 h after intraperitoneal STZ injection, the high blood glucose level, low body weight, and low insulin level were detected gradually in rats from week 1–12. In our study, compared with the control rats, rats with diabetes showed longer EL and shorter time spent in target quadrant. LEV prolonged the target quadrant residence time, while LEV-H achieved a greater improvement in the target quadrant residence time. We confirmed that diabetes-induced cognitive dysfunction occurred in these rats and that LEV improved the behavioral deficits at week 12.

In addition, abnormal cell morphology and contracted cell nuclei were observed in the hippocampus of the diabetic rats. LEV (10 or 100 mg/kg) administered between 72 h and 12 weeks after intraperitoneal STZ injection successfully alleviated the impaired cell morphology and nuclear contraction. High-dose LEV was better than low-dose LEV in ameliorating the cognitive dysfunction and hippocampal damage. These results are similar to those of other studies that LEV improved cognitive function. In a previous study, STZ was injected intra-cerebroventricularly to establish a sporadic AD model, and LEV was administered for 28 days. The results of MWM and passive avoidance tests showed that LEV (100 and 150 mg/kg) significantly alleviated STZ -induced cognitive impairment (Alavi et al., 2022). In another study, Different concentrations of LEV were administered to APP23/MAPT mice and the results suggested that a low dose of LEV alleviated the memory defects (Zheng et al., 2022). In addition to animal studies, a recent randomized clinical trial study also showed that LEV improved executive function and spatial memory ability in patients with AD and epileptiform activity (Vossel et al., 2021). However, the meta-analysis of RCTs reported that LEV increased the risks of psychobehavioural and cognitive adverse events (PBAEs), especially in children. It indicates that LEV might have a detrimental effect on psychobehavioural activity in children, due to their higher susceptibility in this period of life. We should also be cautious about this conclusion, because the evaluations provided by caregivers were subjective and might lack standards to measure behaviour and cognition for DEE (Strzelczyk and Schubert-Bast, 2022). Berk et al. found that in the offspring of LEV group, the alveolar epithelium thickened, the Bowman’s space in renal corpuscles dilated, and the brain cortex irregularly thickened, all indicating that LEV has deleterious effects on the lungs, kidneys and brains of newborns, when 100 mg/kg LEV were intraperitoneally injected before pregnancy of Genetic Absence Epilepsy Rats from Strasbourg rats (Can et al., 2022). These results were not similar to that of our study, which might be due to the prenatal susceptibility of rats and different models. The mechanisms of these results should be further studied to illustrate the deleterious effect of LEV.

One previous study has found that LEV improves the cognitive function of APP transgenic mice, whereas other anti-seizure agents did not (Sanchez et al., 2012). Low-dose LEV (125 mg b.i.d) moderately improved the activity of the hippocampus and cognitive performance, although without significant difference from that of the control (Bakker et al., 2012). Similar results have also been observed in the rat model of age-related memory loss (Koh et al., 2010). Although the mechanisms involved are not very clear, our results are illustrative to some extent.

We observed higher serum TNF-α, IL-1β and IL-6 levels in diabetic rats, which implied that metaflammation occurred in diabetic rats. Our results also showed that LEV decreased them in diabetic rats. High-dose LEV showed the strongest effect in decreasing the level of IL-1β. This effect may be mediated by the inhibition of systemic-inflammatory response. Further, we measured the expression of Iba1 (a marker of microglia) in the hippocampus of STZ-induced diabetic rats in the four groups. Microglia can promote inflammatory response, thus facilitating neuronal apoptosis and brain damage (Voet et al., 2019; Ji et al., 2020; Lynch, 2020). Microglia secrete pro-inflammatory factors during the differentiation into M1. Several anti-inflammatory factors are secreted during differentiation from M1 to M2. The level of Iba1 was significantly increased in diabetic rats. Moreover, high-dose LEV reduced the number of Iba1 positive cells in the CA3 and DG areas of the hippocampus. Niidome et al. (2021) also demonstrated that LEV decreased FosL1 expression and AP-1 activity and suppressed neuroinflammation in BV2 cells pretreated with lipopolysaccharide. The results indicate that LEV can inhibit neuroinflammatory responses in several neurological diseases. Several studies have been conducted to explore whether LEV inhibits inflammation in other diseases. In one of those studies and LEV inhibited microglial activation, suppressed TNF-α and Il-1β levels, and promoted angiogenesis after cerebral ischemia in rats (Yao et al., 2021). Xiong et al. (2020) showed that in lipopolysaccharide-treated microglia cells, LEV treatment significantly decreased inflammatory factors levels and reduced NF-κB and STAT3 protein expressions. In an intracerebral hemorrhage (ICH) rat model, LEV treatment improved neurological function, alleviated brain edema, and decreased NF-κB, JAK2, and STAT3 protein expressions, indicating that LEV can repress early inflammatory responses induced by ICH. Moreover, in AD-related mouse models, microglial alterations are detected. LEV intervention can reverse or prevent brain microglial gene expression in aged human APP transgenic mice (Das et al., 2021). Our results suggested that the proportion of Iba1+ cells was decreased after LEV treatment in the hippocampus.

The results of CCK-8 showed that BV2 cell viability did not change significantly after different glucose concentration treatment. The expeimental principle of CCK-8 is to quantify the number of live cells. The above results indicate that high glucose activated and damaged BV2 cells, but not led to cell death. Lactate dehydrogenase (LDH) is a stable cytosolic enzyme in cells. So LDH test is suitable for cytotoxicity analysis by detecting the activity of LDH in cell culture supernatant. The results illustrated that LDH release was the most prominent in the 50 mM group. Meanwhile, the mRNA expressions of inflammatory genes indicated that 75 mM glucose enhanced the expressions of M2 markers than 50 mM glucose, although it also increased the expressions of M1 markers. Although 25 mM glucose could increase the mRNA expression of IL-1β, TNF-α, and iNOS, this concentration of glucose slightly increased LDH release. Moreover, 25 mM glucose is the upper limit of normal glucose concentration in DMEM. Considering its effects on cell viability, cell morphology, LDH release, and inflammatory factor mRNA expression, 50 mM was determined as the optimum glucose concentration to establish high glucose-induced inflammation model in BV2 cells.

To further confirm the effect of LEV on inflammation *in vitro*, BV2 cells were cultured in a high glucose medium to establish an *in vitro* cell model. In high glucose-stimulated BV2 cells, the same inflammatory response was observed as that found *in vivo*. In the *in vitro* cell model (BV2 cells and the cell supernatant), LEV 50 µM reduced IL-1β, TNF-α and iNOS mRNA and protein levels after 50 mM high glucose stimulation. Moreover, IL-10 and CD206 mRNA expressions were upregulated in the LEV 50 µM treatment group. In addition, 50 µM LEV decreased IL-1β and TNF-α levels but increased IL-10 level in BV2 cells with high glucose-induced inflammation. Furthermore, 50 µM LEV increased the proportion of iNOS+/Iba1+ cells and decreased the proportion of CD206+/Iba1+ cells, suggesting that LEV alleviated high glucose-induced microglial polarization. However, the anti-polarization effect of LEV was weakened by anisomycin, indicating that the anti-polarization effect of LEV in BV2 cells with high glucose-induced inflammation might be related to the MAPK signaling pathway.

MAPKs regulate diverse in neuroinflammatory responses in brain dysfunction (Das et al., 2012). MAPKs can activate the ERK, JNK, and p38 pathways (Liu et al., 2019; Hammouda et al., 2020; Mai et al., 2020), and the downstream genes to promote the release of pro-inflammatory factors (Mai et al., 2020). The phosphorylation of JNK is elevated in the brain tissue of AD and the hippocampus of APP/PS1 transgenic mice (Bomfim et al., 2012). To elucidate the signaling pathways involved in the anti-polarization effect of LEV on microglia after glucose stimulation, we measured the expression of MyD88, TRAF6, TAK1, p-TAK1, JNK, p-JNK, p38, p-p38, NF-κB p65, p-NF-κB p65 in the control group, high-glucose group, and LEV 50 µm treatment group by Western blotting. High glucose enhanced the expression of MyD88 and TRAF6 and the phosphorylation of TAK1, JNK, p38, both then inhibited by LEV 50 µm. This finding implied that LEV-mediated cognitive enhancement was mediated via inhibiting abnormal phosphorylation of MAPKs. NF-κB, which is activated by endotoxin, bacteria, cytokines, and tumor antigens, can migrate to the nucleus to promote pro-inflammatory protein expression (Liu J. et al., 2018; White et al., 2020).

To characterize the mechanism underlying high glucose-induced microglial polarization, NF-κB p65 and p-NF-κB p65 expressions were detected via Western blotting. Our results showed that the expression of NF-κB p65 and p-NF-κB p65 was increased in high glucose-induced microglia. However, 50 µm LEV markedly attenuated the STZ-induced phosphorylation of NF-κB-p65, indicating that the anti-polarization of LEV on microglia occurred via inhibition of NF-κB signaling activation. Moreover, the anti-inflammatory and anti-polarization effects of LEV were partly reversed in BV-2 cells induced by high glucose. Thus, our results indicated that LEV improved cognitive function through the JNK/MAPK/NF-κB signaling pathways.

However, this study has several limitations. The diabetic rat model was established using single-dose STZ injection to induce insulin deficiency and hyperglycemia. The mechanism of cognitive impairment related to insulin resistance was not studied in our research. Despite these limitations, the study has its own strengths. First, this is the first study to characterize the effect of LEV on the cognitive function of STZ-induced diabetic rats. Second, we also investigated the therapeutic mechanism of LEV from the perspective of neuroinflammation and microglial polarization, thus providing a better understanding of diabetic cognitive impairment.

In conclusion, LEV is efficacious in improving cognitive function and hippocampal injury in STZ-induced diabetic rats. The effect is associated with a reduction in inflammatory cytokines. Our findings suggested that LEV exerts its protective effect by preventing microglial polarization and inducing microglial M2 transformation through the JNK/MAPK/NF-κB signaling pathways.

## Data Availability

The original contributions presented in the study are included in the article/Supplementary Material, further inquiries can be directed to the corresponding author.
